# Effects of platelet rich plasma and chondrocyte co-culture on MSC chondrogenesis, hypertrophy and pathological responses

**DOI:** 10.17179/excli2017-453

**Published:** 2017-07-16

**Authors:** Rouhallah Ramezanifard, Mahboubeh Kabiri, Hana Hanaee Ahvaz

**Affiliations:** 1 Department of Biotechnology, College of Science, University of Tehran, Iran; 2 Department of Stem Cell Biology and Department of Molecular Biology, Stem Cell Technology Research Center, Tehran, Iran

**Keywords:** mesenchymal stem cell, AC:MSC co-culture, PRP, hypertrophy, angiogenesis, inflammation

## Abstract

Regarding the inadequate healing capability of cartilage tissue, cell-based therapy is making the future of cartilage repair and regeneration. Mesenchymal stem cells (MSC) have shown great promise in cartilage regeneration. However, a yet-unresolved issue is the emergence of hypertrophic and pathologic markers during *in vitro* MSC chondrogenesis. Articular chondrocytes (AC) can suppress the undesired hypertrophy when co-cultured with MSC. On the other hand, platelet rich plasma (PRP), is considered potentially effective for cartilage repair and in-vitro chondrogenesis. We thus aimed to harness chondro-promotive effects of PRP and hypertrophic-suppressive effects of AC:MSC co-culture to achieve a more functional cartilage neo-tissue. We used PRP or conventional-differentiation chondrogenic media (ConvDiff) in MSC mono-cultures and AC:MSC co-cultures. We assessed gene expression of chondrogenic and hypertrophic markers using real-time RT-PCR and immunostaining. Alkaline-phosphatase activity (ALP) and calcium content of the pellets were quantified. We also measured VEGF and TNF-α secretion via ELISA. We showed PRP had higher chondrogenic potential (in mRNA and protein level) and hypertrophic-suppressive effects than Conv-Diff (mRNA level). Co-culturing reduced ALP while PRP increased calcium deposition. In all four groups, TNF-α was down-regulated compared to MSC controls, with co-cultures receiving ConvDiff media secreting the least. Meanwhile, the only group with increased VEGF secretion was PRP-mono-cultures. We observed synergistic effects for PRP and AC:MSC co-culture in enhancing chondrogenesis. Inclusion of AC reduced hypertrophic markers and angiogenic potential in PRP groups. We thus propose that combination of PRP and co-culture would favor chondrogenesis while alleviate but not totally eradicate undesired hypertrophic and pathologic responses.

## Introduction

Articular cartilage tissue creates a smooth surface which facilitates joint movement (Becher et al., 2010[8]). Once injured, this tissue has limited ability to repair itself which in turn can lead to lesion progression and finally osteoarthritis (Ahern et al., 2009[2]; Bedi et al., 2010[9]). The lack of efficient pharmacologic treatments along with inherent inefficiency of the healing process has extended the cell-based methodologies for cartilage repair either by replacement or reparative procedures.

Autologous chondrocytes (AC) have been successfully used for *in vivo* grafting into chondral lesions (Acharya et al., 2012[1]). They are able to produce stable and non-mineralized cartilage tissue. However, it is difficult to obtain enough of chondrocytes which is due to the small biopsied sample, the resultant donor tissue morbidity and limited chondrocyte proliferation potential. Chondrocyte dedifferentiation after extensive *in vitro* propagation is another drawback (Cooke et al., 2011[13]). On the other hand, mesenchymal stem cells (MSC), as an alternative autologous source, have excellent potential for chondrogenesis. These cells could be easily obtained from different sources with bone marrow and adipose tissue being the most studied, available and promising sources (Shafiee et al., 2016[37]). MSC's self-renewal potential is superior to chondrocytes in terms of speed, passage number and maintenance of immuno/phenotypic characteristics during *in vitro* propagation (Farrell et al., 2011[17]; Marquass et al., 2011[28]; O'Sullivan et al., 2011[32]; Acharya et al., 2012[1]). Moreover, MSCs secrete several cytokines and growth factors with immuno-modulatory effects, which enable them with a unique therapeutic role in osteoarthritis or rheumatoid arthritis diseases (Mamidi et al., 2016[27]). However, regarding the complex molecular mechanism of chondrogenesis, strong inductive and directive signals are required to differentiate MSCs into chondrocytes (Bian et al., 2011[10]). Yet unresolved drawbacks with MSC, including unwanted hypertrophy reminiscent of endochondral ossification and emergence of some cartilage pathologic markers such as angiogenic features, can restrain their robustness (Dickhut et al., 2009[14]). This implies that more research is needed in order to prevent or minimize such impediments via developing new protocols that induce maximal chondrogenesis with minimal hypertrophy, as is desirable for cartilage repair interventions.

Autologous platelet rich plasma (PRP) as a blood supply with high concentration of the platelets (Smyth et al., 2012[39]) is considered a valuable source for healing process enhancement in various cases, being clinically in use for tissue repair purposes (Boswell et al., 2012[12]; Lee et al., 2012[25]). Upon activation, alpha granules as a rich source of numerous growth factors such as transforming growth factor beta (TGF-β), vascular endothelial growth factor (VEGF), platelet derived growth factor (PDGF), insulin-like growth factor (IGF) and basic fibroblast growth factor (bFGF) release their components into the surrounding cell environment (Zhu et al., 2013[47]). This wide variety of growth factors and cytokines contribute to multifaceted roles of PRP. After addition to *in vitro* cultures, PRP can increase cell proliferation (Kon et al., 2010[24]; Filardo et al., 2012[18]), enhance stem cell differentiation (Spreafico et al., 2009[42]; Smyth et al., 2012[39], 2013[40]) and raise matrix production (Akeda et al., 2006[4]). Also, PRP has been used for regenerative injection therapy of osteoarthritis causing symptomatic relief in early osteoarthritis and improving clinical outcome (Filardo et al., 2012[18]; Patel et al., 2013[34]). PRP has mostly gained its popularity as an adjunct to a variety of surgical procedures by reducing postoperative complications, pain and improving tissue healing (Yuan et al., 2012[46]). Clinically, PRP in conjunction with MSC or AC is applied to partial, full, osteochondral and osteoarthritic defects (Zhu et al., 2013[47]). Yet, depending on the source, preparation protocol and final growth factor and cytokine content of PRP, different degrees of success have been achieved and still up-coming pathologies remain controversial. Of the numerous cytokines and growth factors present in PRP lysate, parts of them are conducive to maintain the phenotype of chondrocytes, others are not (Zhu et al., 2013[47]). Meanwhile, PRP is proved to have both pro- and anti-inflammatory effects (Anitua et al., 2015[6]).

The classic chondrogenic protocols involving serum-free media supplementation with dexamethasone and transforming growth factor (TGF-β), though promising in up-regulating cartilage specific matrix molecules, can also induce hypertrophic and catabolic markers (Shen et al., 2014[38]). In order to improve chondrogenic differentiation protocols and the resultant MSC derived cartilage neo-tissue, enhanced chondrogenesis of MSCs cocultured with chondrocytes has been emphasized which is demonstrating higher cartilage specific marker expression in the mixed groups compared with monocultures as well as diminished expression of hypertrophic markers (Bian et al., 2011[10]; Babur et al., 2015[7]). Development of a reliable chondrogenic differentiation protocol without occurrence of pathologic or osteoarthritic relevant phenotype will prosper clinical stem-cell based cartilage repair strategies.

MSC have established trophy effects towards various cell types (Wu et al., 2012[44]; Kabiri et al., 2013[22]). MSC can help chondrocytes and get affected by chondrocytes. MSC from adipose tissue are shown to effectively reduce hypertrophy and dedifferentiation of chondrocytes, likely via HGF secretion (Maumus et al., 2013[29]). In direct co-culture with primary MSC, articular chondrocytes can suppress their hypertrophic differentiation and prevent matrix calcification (Acharya et al., 2012[1]). We thus hypothesized that the immunomodulatory effects of MSC, trophic effects of AC and the rich bioactive agents of PRP may have synergistic effects on chondrogenesis with enhancing matrix production and dampening hypertrophic differentiation.

Although both strategies (applying PRP and co-culture with AC) have already shown to be promising in this regard, there seems to be no previous study employing the two in combination. Also their effects, alone or in combination, on cartilage pathologic marker suppression such as inflammation and angiogenesis, has not yet been addressed. Thus, in the current study we aimed to evaluate if the unfavorable outcomes, usually concomitant with *in vitro* chondrogenesis, such as hypertrophic differentiation, angiogenesis and inflammation responses, would get minimized using the combination of these two strategies. To do that, we made AC-MSC pellet co-cultures and endowed them with PRP and compared their chondrogenic, hypertrophic and pathologic marker expression with MSC mono-cultures and conventional differentiation methods.

## Materials and Methods

### Cell isolation and culture

MSC were isolated from adipose tissue obtained from cosmetic liposuction surgeries from 3 different donors (aged 18-40, Taleghani Hospital, Tehran, Iran) with their full consent and willingness according to Stem Cells Technologies Research Center Ethics guidelines. Adipose resident stem cells were isolated as described in details previously (Shafiee et al., 2016[37]). Cells were expanded and subcultured till passage 6.

Articular chondrocytes (AC) were isolated from patients with grade 3 or 4 articular cartilage damage undergoing knee replacement surgery, after obtaining their consent. The cartilage tissue was cut out from the damaged joints and cells were isolated as previously described (Babur et al., 2015[7]). AC were passaged at 80 % confluency and cells at passages 4-6 were used for the main experiments.

### Chondrogenic treatments

Chondrogenic cultures were formed as cell pellets in 15 ml polypropylene conical tubes containing 2.5×10^5 ^cells per pellet in all groups. In co-cultures, MSC were mixed in the same pellet with AC at a 10:1 ratio and cultured for two weeks. Following pellet formation, in both mono-cultures and co-cultures, media were exchanged with either conventional chondrogenic media containing high-glucose DMEM (Invitrogen) supplemented with ITS, proline, pyruvate, ascorbate-2-phosphate, dexamethasone (all from Sigma), TGF-β1 (Peprotech) and pen/strep with concentrations described previously (Babur et al., 2015[7]), hereafter referred to as “Conventional Differentiation, Conv-Diff” group or high-glucose DMEM supplemented with 10 % PRP and pen/strep referred to as “PRP” group. Parallel pellets without any AC (MSC mono-cultures) were also treated in the same way to figure out the role of AC-MSC co-culture. Thus the four groups were named as “mono-PRP” and “mono-ConvDiff” for MSC mono-cultures treated with either PRP or conventional chondrogenic differentiation medium and “Co-PRP” and “Co-ConvDiff” for groups containing the 10:1 MSC:AC. All pellets were maintained in incubator at 37 ˚C in 5 % CO_2_ for two weeks with media exchange twice a week. All the analyses were performed at day 14. 

### PRP preparation

Whole blood with an anti-coagulant agent was collected from volunteer donors. After centrifugation (3200 rpm, 10 min) the upper plasma phase was collected and centrifuged again at 4000 rpm for 15 minutes. The concentrated platelets were dispensed in platelet poor plasma (PPP), activated with one tenth volume of 10 % CaCl_2_ and incubated overnight at 4 °C followed by filtration through 0.2 μm sterile filter on the following day. The resultant activated PRP was then stored at -70 °C and used up to one month (Pakfar et al., 2017[33]). In order to minimize variations, activated PRP from three different individuals were mixed and used for main experiments.

### Real-Time RT-PCR

Expression pattern of major chondrogenic and osteogenic markers was assessed using Real-Time RT-PCR after two weeks of chondrogenic induction. Total RNA was extracted using RNA extraction kit (CinnaGen). cDNA was synthesized using a cDNA kit according to manufacturer instructions (Takara). The resulted cDNA were evaluated in 40 rounds of Real-Time PCR reaction (Corbett) and data were analyzed by Rotor Gene Q software. Primer sequences are demonstrated in Table 1(Tab. 1). Using ∆∆Ct method, the expression of each gene was normalized against β-actin as internal control and estimated in Co-PRP group relative to Co-ConvDiff one and also in mono-PRP group relative to mono-ConvDiff one in order to underline the effect of PRP treatment in either mono or co-culture platform and have them compared with Conv-Diff media classically used in co-culture experiments.

### Histological analysis (Alcian Blue staining and immunocytochemistry)

After two weeks of induction, the pellets were fixed in cold 4 % paraformaldehyde, embedded in paraffin, sectioned (5 µm thick) and used for immunocytochemistry and Alcian Blue staining. After deparaffinization and rehydration, antigen retrieval was carried out using sodium citrate. The immunostaining was carried out using type II Collagen (Col-II, 1:20 diluted) or type X Collagen (Col-X, 1:80 diluted) rabbit anti-human primary antibodies (Abcam) at 4 °C overnight and stained with PE-conjugated goat anti-rabbit secondary antibody (1:100 diluted, 1 h at 37 °C, eBioscience). For GAG staining, the sections were stained with Alcian Blue 8GX (Roth) as previously described (Shafiee et al., 2011[36]). Sections were then photographed using a fluorescent microscope (Nikon) assisted with an IP-camera (Genucam).

### Alkaline phosphatase activity

Alkaline phosphatase (ALP) activity as a hypertrophic differentiation marker was assessed by adding 200 µL of RIPA buffer to each pellet at day 14 of culture. Cell lysate was then centrifuged at 15000 rpm, 4 °C for 15 min. The supernatant was collected and ALP activity was evaluated with an ALP assay kit (Parsazmun) according to the manufacturers' instruction. Finally, enzyme activity was normalized against total protein (BCA kit, Life Technologies).

### VEGF and TNFα secretion assessment

In order to evaluate the effect of differentiation treatments and the culture type on secretion of TNFα, a marker of pro-inflammatory responses, by chondrogenic cells at day 14 the media were collected and quantitatively assayed for TNFα secretion. Since PRP itself has some endogenous amounts of TNFα, in PRP groups the media were exchanged with basal media (High Glucose DMEM, 1 % ITS) for two days before media collection and further TNFα assay by ELISA kit (Peprotech). In brief, capture antibody was added to the strips after dilution to the working concentration and incubated overnight as per manufacturer's instruction. After washing with 0.05 % Twin 20 in PBS, blocking buffer, samples or standards and detection antibody were added successively with a washing process following each step. TNFα was calculated by comparison with standard curve. Finally, the data were normalized against total protein content measured by BCA assay kit (Life Technologies).

Likewise, for VEGF assay, culture media were collected and used for ELISA using mini ELISA development kit (Peprotech) according to the manufactures' instruction. HRP activity of the detection antibody was assayed using ABTS agent and read at 405 nm. The wavelength of 650 nm was used as reference. Similar to TNFα assay, the two-day basal medium was collected and used for ELISA.

### Statistical analyses

All tests were carried out in triplicate. Data are shown as mean ± SD. For data comparison, One-way analysis of variance (ANOVA) was used and 0.05 was considered as p-value for statistical significance. All statistical analyses were conducted by SPSS software, version 11.0.

## Results

### Chondrogenic effects of PRP in mono- and co-cultures

**Figure 1:**
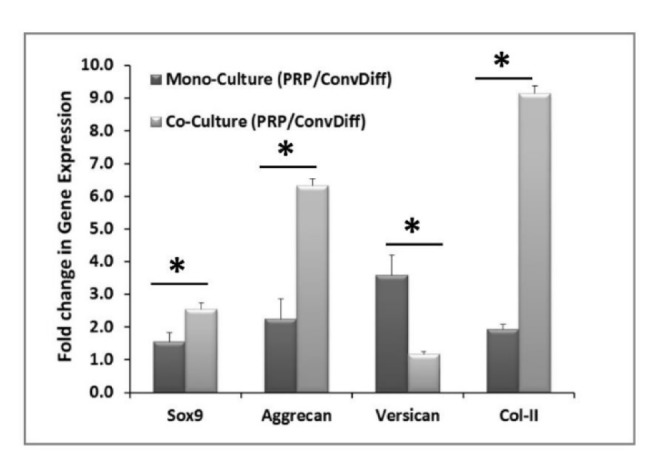
Chondrogenic gene expression after two weeks of induction. The data are relative gene expression obtained by ∆∆Ct calculations for each gene in PRP group relative to Conventional-Differentiation control (Conv-Diff). β-actin is used as internal control. * indicates significant difference between mono- and co-cultures (p<0.05). Except for Versican in the co-culture group, the rest of the markers showed upregulation in PRP relative to Conv-Diff.

After two weeks of induction with either PRP or conventional chondrogenic differentiation media under both mono- and co-culture platforms, gene expression was assessed in mRNA and protein level. Relative gene expression was assessed for a panel of chondrogenic markers using Real Time RT-PCR by calculating ∆∆Ct of PRP treated co- and mono-culture groups compared against the respective conventional differentiation co- and mono-culture groups, respectively, for each specific gene. As illustrated in Figure 1(Fig. 1), all chondrogenic markers examined, including Sox-9, Aggrecan, Versican and Col-II were significantly increased (from 1.2 to 9.1 folds) in PRP groups compared to the conventional differentiation control groups (p < 0.05). Except for Versican, this increase was more pronounced (with statistically significant difference, p<0.01) in co-cultures than in mono-cultures. Which means that PRP has acted synergistically with co-culturing method in enhancing chondrogenic differentiation of MSC.

**Figure 2:**
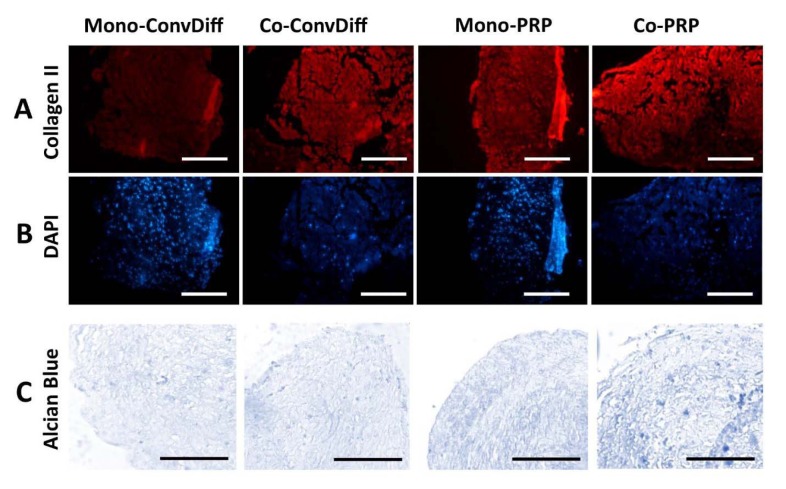
Chondrogenesis evaluated by ECM production and deposition. A: Expression of Col-II in samples stained with PE-conjugated anti-Col-II antibody; Scale bars: 400 µm. B: Same sections stained with DAPI, placed beneath the pertinent Col-II micrograph. C: Sections stained with Alcian Blue marking GAG production in the ECM of the constructs; Scale bars: 400 µm

In terms of protein expression and ECM secretion and based on the numerous micrographs analyzed for each treatment, we observed that co-ConvDiff and mono- and co-PRP groups had deposited more Col-II (Figure 2A,B(Fig. 2)) as compared to mono-ConvDiff. Though images can just be qualitatively judged, obviously the least Col-II secretion was observed in mono-ConvDiff group. Although no additive effect can be inferred for PRP and co-culture, the images show that both PRP and co-cultures are privileged in enhancing Col-II production which is definitely a determinant factor in normal physiology and function of the cartilage. In Alcian Blue stained sections, all samples were rather similar with co-PRP being a bit more stained (Figure 2C(Fig. 2)). These would express that all treatments were almost equally effective in GAG deposition.

### Hypertrophic differentiation of MSC under mono- and co-cultures with PRP or conventional chondrogenic regimens

**Figure 3:**
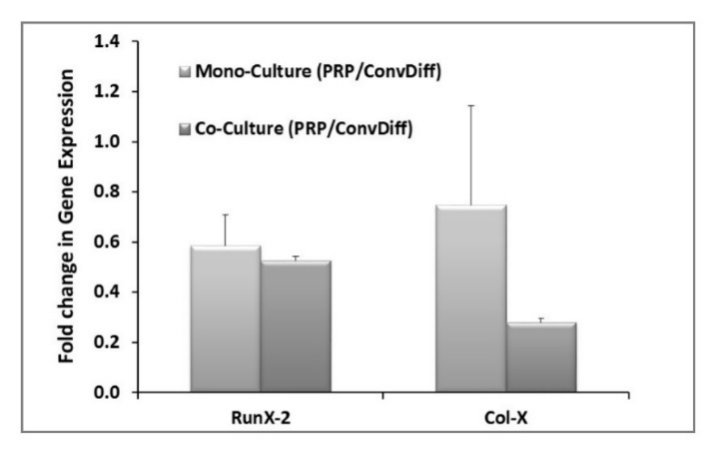
Hypertrophic gene expression after two weeks of induction. The data are relative gene expression obtained by ∆∆Ct calculations for each gene (RunX-2 and Col-X) in PRP group relative to Conventional-Differentiation control (Conv-Diff). β-actin is used as internal control.

**Figure 4:**
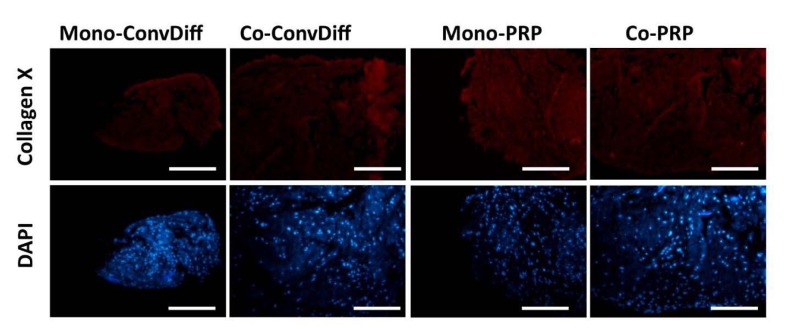
Hypertrophy evaluated by Col-X deposition in ECM. A: Expression of Col-X in samples stained with PE-conjugated anti-Col-X antibody; B: Same sections stained with DAPI. Scale bars: 400 µm

The expression of hypertrophic markers (Col-X and Runx-2) in terms of mRNA expression level was decreased in both PRP groups (co- and mono-) when compared to the corresponding Conv-Diff (co- and mono-) groups (Figure 3(Fig. 3)). Though there was no difference between mono- and co-cultures in RunX-2 expression, the maximal prohibitory effects on Col-X expression was observed in Co-PRP group, suggesting that PRP and co-culture had additive effects in suppressing Col-X mRNA. Suppression of hypertrophic differentiation is of significant importance as it could prevent successive tissue ossification and cartilage abrasion. We also examined the Col-X protein via immunostaining of pellet sections. Regarding the point that photographs were captured with the same light exposure, from the light intensity of the micrographs we can deduce that in all groups less type X collagen protein was deposited relative to type II collagen, recalling the effectiveness of chondrogenesis in all groups. Meanwhile, in both mono- and co-cultures receiving Conv-Diff media, less Col-X production is noticeable than in PRP groups (Figure 4(Fig. 4)).

**Figure 5:**
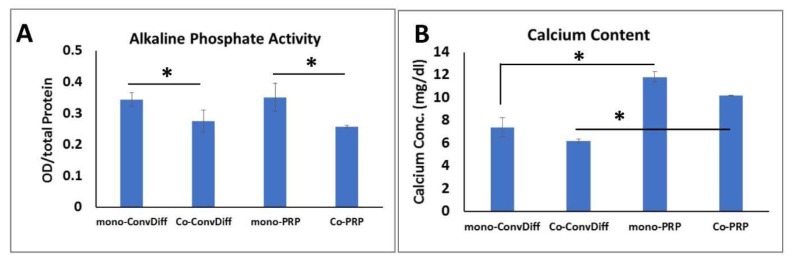
ALP activity and calcium content as markers of hypertrophic differentiation. A: Normalized ALP activity, B: Calcium deposited in the pellets after two weeks of chondrogenic induction. * indicates significant difference (p<0.05). Values are mean ±SD.

ALP as another hypertrophic marker showed significantly reduced activity in co-cultures (both PRP and Conv-Diff) relative to mono-cultures (Figure 5A(Fig. 5)). There was no significant difference in each mono- and co-culture regimens between PRP and conventional differentiation groups. In fact it is the presence of AC that has decreasing effect on ALP activity.

Calcium deposition is considered a hallmark of osteogenically differentiated cells or hypertrophic maturation of chondrogenic cells. Among the four different treatments, we observed the highest calcium deposition in mono-PRP group (Figure 5B(Fig. 5)). This result was rational, since along with chondro-promotive factors present in PRP, growth factors such as BMP can at least partly induce osteogenesis. This effect was somehow abandoned in co-PRP group, which imparts the suppressive effects of chondrocytes on osteogenic cues of PRP. There was a subtle decreased deposition of calcium in co-ConvDiff group relative to mono-ConvDiff. Deposited calcium was significantly higher in mono-PRP compared to mono-ConvDiff and in co-PRP compared to co-ConvDiff.

### TNFα and VEGF production and secretion

**Figure 6:**
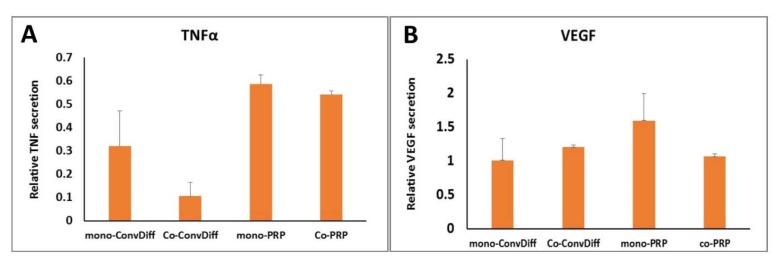
Pathologic markers of chondro-induced pellet cultures. A: TNF-α secretion quantified by ELISA; Bars indicate secreted TNF into the media relative to that in undifferentiated MSC controls. B: VEGF secretion quantified by ELISA, expressed as values relative to MSC controls. Values are mean±SD.

TNFα is a cytokine involved in systemic inflammation. Although it is largely produced and secreted by activated macrophages, it can also be synthesized by many other cells and tissues such as adipose tissue (Kilroy et al., 2007[23]). In order to assess the inflammatory potential of differentiated cells, TNFα secretion was measured quantitatively as shown in Figure 6A(Fig. 6). All four chondrogenic approaches examined showed reduced TNFα secretion in comparison to undifferentiated MSC controls (40 %-90 %). Though PRP is mostly privileged for its anti- inflammatory potentials rather than pro-inflammatory ones, the cells in Co-ConvDiff group showed the least TNFα secretion. However, when PRP was also included co-culturing did not show such suppressing effect.

VEGF is a signal protein stimulating vasculogenesis and angiogenesis. Having considerable amounts of VEGF, PRP has proved angiogenic potential. Though suitable for wound healing and tissue remodeling, it is not desired when it comes to cartilage regeneration. Herein we observed about 50 % increase in VEGF secretion in mono-PRP compared to MSC controls (Figure 6B(Fig. 6)). However Co-PRP was equal to Co-ConvDiff, recalling that co-culturing has dampened the angiogenic potential of PRP.

## Discussion

Chondrocytes are the ideal cell type to repair cartilage lesions, however the required *in vitro* expansion procedure to obtain sufficient chondrocytes, usually induces chondrocyte dedifferentiation and altered or loss of functionally. So MSC stand out as a promising alternative cell source for cartilage repair. However, at the late stages of MSC chondrogenesis, hypertrophic differentiation and pathologic responses can overwhelm MSC applicability. Lately, co-culture of AC and MSC has shown to mitigate the above mentioned issues, while PRP has been used in order to further enhance the chondrogenesis. We thus assessed the functionality and the combinatory effects of PRP with AC:MSC co-culture using pellet cultures, aiming to fetch up optimal chondrogenesis while minimizing undesired hypertrophy and pathologic responses.

PRP has key molecular sources involved in tissue repair and regeneration. However, reports on the effects of PRP can be considered of the most diverse and somehow contradicting ones. Such variety in effects can stem from different preparation of PRP and variable growth factors present in PRP obtained from different individuals. To minimize such variations we made a pool of activated PRP from three different donors and utilized it for the main experiments. In line with previous studies we observed up-regulation of chondrogenic markers in all treatments (Drengk et al. 2009[15]). Collectively in both mono- and co-culture strategies, PRP showed to be a stronger inducer of chondrogenesis compared with conventional differentiation media containing TGF-β as the only present growth factor. The highest impact was observed in Aggrecan and Col-II mRNA and Col-II protein, wherein PRP proved higher chondrogenic markers expression. This can be attributed to the presence of factors such as bFGF along with TGF-β present in PRP, acting in synergy, which has led to increased chondrogenesis in PRP groups (Böhme et al., 1995[11]; Fischer et al., 2010[19]). Such synergistic effect is even more pronounced in co-culture groups. That is, Sox-9 as an upstream and early regulator of cartilage matrix production along with Aggrecan and Col-II were significantly upregulated in co-cultures compared to mono-cultures. The enhancive effect of co-culture on Col-II protein was only observable for Conv-Diff treatments with co-ConvDiff secreting more Col-II compared to mono-ConvDiff, but not in PRP mono- and co-cultures, in which both groups were rather similar in producing Col-II protein. Nevertheless, in spite of all chondro-promotive effects observed in this study and proved elsewhere, PRP has osteogenic and angiogenic effects as well (Malhotra et al., 2013[26], Xie et al., 2014[45]). Thus, a more robust survey is required to determine whether such phenotypes can lead to pathologic outcomes, hindering the usefulness of the constructed neo-tissue in cell-therapy approaches. As such, we stepped forward to assess the hypertrophic, angiogenic and inflammatory responses in pellet constructs already proved to have good enough chondrogenic properties.

Occurrence of matrix mineralization characterized by alkaline phosphatase activity and cell-construct calcification is correlated with the formation of an undesirable transient cartilage phenotype. However regarding the osteoinductive capacity of PRP, it was not unlikely to witness up-regulation of deposited calcium in PRP groups (about 40-50 %) compared to Conv-Diff treatments, with presence or absence of AC having not a considerable effect on this phenomenon. However, in contrast to calcium deposition, ALP activity seemed to get influenced more by the presence of chondrocytes rather than growth factor supplementation of the medium.

About 20-25 % decrease in ALP activity was observed in co-cultures (both Conv-Diff and PRP) in comparison to monoculture groups. Collectively speaking, the best treatment with the least calcium deposition and ALP activity was co-ConvDiff, while mono-PRP would rank as the last one. This was also inferable from Col-X protein secretion. PRP seemed to be more efficacious in suppressing hypertrophic markers in mRNA level. While AC:MSC coculture proved more efficient in reducing Col-X secretion and deposition and ALP activity. In line with our study Fischer et al. reported down regulation of Col-X/Col-II mRNA ratio, the amount of Col-X deposition and the ALP activity in co-cultures likely via secretory factors present in AC-conditioned media. Though, they observed even more pronounced effects in direct co-culture systems (Fischer et al., 2010[19]).

In order to obtain a healthy stable cartilage neo-tissue from MSC, the molecular events that basically trigger terminal hypertrophic differentiation should be suppressed. This is particularly challenging since MSC condensation in micro-pellet cultures, as a mimic to developmental conditions of cartilage formation, naturally reflects endochondral ossification. The direct interaction between MSC and chondrocytes in pellet co-cultures is proved to have inhibitory effects on terminal hypertrophic differentiation of MSC during *in vitro* chondrogenesis (Babur et al. 2015[7]). This has been attributed to articular chondrocytes teaching MSC to obtain and maintain chondrocyte phenotype either via direct heterotypic cell-cell interactions or soluble factors secreted by them (Acharya et al., 2012[1]). We thus mixed MSC and chondrocytes in a single pellet to provide a direct co-culture system. The two cell types were mixed and cultivated with 10 % PRP supplement (as a cocktail of chondrogenic factors) or under conventional chondrogenic medium containing TGF-β, since it is needed both to promote MSC chondrogenesis and to inhibit chondrocyte dedifferentiation. Dedifferentiated chondrocytes not only tend to lose their ability to deposit collagen and proteoglycan but also lean to hypertrophic phenotype (Bian et al., 2011[10]). The detailed mechanism underlying these effects is not well unravelled. However, the soluble factors secreted by both cell types and the direct cell-cell contact are known as potent means to enhance chondrogenesis and mitigate hypertrophic and pathologic development of MSCs. Numerous factors have been introduced as mediators in the MSC-AC cross talk. Of soluble cytokines PTHrP and FGF-2 are important factors involved in this effect (Fischer et al., 2010[19]). Using a rat model and a co-culture between MSC and cartilage chips, Ahmed et al. suggested VEGF, MMP-13 and tissue inhibitor of MMP-1 and 2 as factors involved in the regulation of hypertrophic marker (Ahmed et al., 2007[3]). Though such proposed factors were not specifically investigated in our study, we observed that the interaction between the two cell types in the co-culture system in combination with the various factors in PRP resulted in a more robust chondrogenesis.

Clinically, transplantation of MSC to articular defects, especially for alleviating arthritis symptoms is highly appreciated. However, MSCs may secrete high concentration of pro-angiogenic cytokines which can overwhelm their cartilage therapeutic outcomes (Duffy et al., 2009[16]). Even pre-differentiated chondrogenic MSC constructs transplanted *in vivo* have shown to undergo vascular invasion and microossicle formation (Pelttari et al., 2006[35]). Vascularization occurs as a response to VEGF production in the neo-construct. Cartilage vascularization is undesired as it can recruit inflammatory cells to the tissue with following interference with healing processes. Also, angiogenesis is highly correlated with late stages of endochondral ossification and osteogenesis (Gerber et al., 1999[20]). As such anti-angiogenic factors are highly advised to inhibit such pathways. Thus we examined the effects of RPP and AC:MSC co-culture and their combination on the secretory growth factors of differentiated MSC.

Despite all favorite attributes of PRP, it can promote angiogenesis that in the case of cartilage tissue is highly undesired. Our result showed that the presence of AC could have counteracted the angiogenic effects of PRP, as in mono-ConvDiff, Co-ConvDiff and Co-PRP groups there was no elevated levels of VEGF as compared to MSC controls. However mono-PRP treatment showed about 50 percent increase in VEGF secretion. This suggests that upcoming problems after neo-construct cartilage implantation pertaining to vasculogenesis can be diminished by utilizing a co-PRP approach. In fact co-PRP has angio-suppressive effects as deduced from comparisons with mono-PRP. This is particularly valuable as in cartilage defect therapies using PRP, usually an innocuous antagonist or VEGF blocking agent such as anti-VEGF antibody is employed (Nagai et al., 2010[31]; Mifune et al., 2013[30]).

Production and secretion of inflammatory factors such as TNFα might lead to cartilage matrix degradation which is regulated through MAPK and canonical NF-κB pathways via inducing the activity of aggrecanases and MMPs (Wojdasiewicz et al., 2014[43]). Pro-inflammatory cytokines are particularly associated with pathogenesis of osteoarthritis since they can mediate the catabolic pathways in cartilage tissue and trigger recruiting pro-inflammatory cells (Spaková et al., 2012[41]). As such suppression of the related pathways is highly desired in cartilage tissue engineering. PRP has proved anti-inflammatory effects and is widely used to alleviate inflammatory symptoms of arthritis (Zhu et al., 2013[47], Xie et al., 2014[45]). In all our treated groups we observed reduced expression of TNFα as compared to MSC controls. Which means that pre-differentiation of MSC either via traditional chondrogenic protocols or PRP supplementation can favor down-regulation of inflammatory pathways after cell transplantation to the cartilage defect site. In fact the anti-inflammatory effects associated with postoperative interventions using PRP can be explained by the initial inhibition of macrophage proliferation (Zhu et al., 2013[47]). Although PRP is mostly known with its anti-inflammatory properties, some studies show that it can also have pro-inflammatory cytokines such as IL-1β and IL-6 (Zhu et al., 2013[47]; Xie et al., 2014[45]). For example the effects of PRP on tendon fibroblasts is reported to be induction of inflammatory responses and activation of oxidative stress pathways (Hudgens et al., 2016[21]).

The pro-inflammatory cytokines in some PRP preparations have been attributed to the presence or inclusion of leukocytes (of the buffy coat) in the final cell/platelet preparation (Anitua et al., 2015[6]). Herein, we observed the most down-regulation of TNFα in co-ConvDiff group, while there was no significant difference among the other three groups. Which means that AC:MSC co-culture not only reduces hypertrophic differentiation but also can help dampening inflammatory pathways. Such intensified anti-inflammatory effects in co-ConvDiff is not observed in co-PRP group, which might be explained by the AC cytokines being diluted in the diverse pool of PRP cytokines. In order to counteract or inhibit pro-inflammatory cytokines and reduce joint catabolism after cell therapy of a defected cartilage, IL-1 receptor antagonist protein as well as anti-inflammatory interleukins such as IL-4 and IL-10 can be applied (Andia and Maffulli, 2013[5]).

PRP contains hundreds of different biologically active molecules. Chondrocytes and even MSC can add to this milieu by secreting additional bioactive molecules. The net effect on cell fates is thus very unpredictable which will even get more complicated regarding the numerous PRP preparation protocols and formulations, the way of activation, the endogenous heterogeneity of MSC and AC cultures and the age and other complications pertinent to cell and blood donors. To mitigate such variations and the uncertainty in the biological effects we made a pool of PRP from three different blood donors and used three MSC and two AC donors. Although variations in results did exist, the overall trends in the investigated parameters were rather similar. Meanwhile this work had several limitations as the neo-cartilage tissues obtained via any of the treatments might respond differently *in vivo*. Therefore, a complete examination of the chondrogenic, hypertrophic, angiogenic and inflammatory markers are needed before making any judgment about the prominence of any of these treatments to reach the optimal protocol with suitable tissue quality. Except for the two collagens tested, we did not evaluate gene expression in protein level, while it is so likely that the changes in mRNA level do not correspond to changes in proteins.

## Conclusion

By exploiting the benefits of co-culture systems of MSC with chondrocytes in pellet culture, we sought to evaluate whether chondro-promotive effects of PRP and hypertrophy-prohibitive effects of AC:MSC co-culture could be combined with synergistic consequences. Regarding problems associated with MSC transplantation to cartilage lesions plus inevitable hypertrophic differentiation of MSC during *in vitro* chondrogenesis, we proposed that by pre-differentiation of MSC using PRP and AC:MSC co-culture, the chondro-promotive effects of PRP and hypertrophy-suppressive effects of AC:MSC co-culture might sum up in order to obtain a stable articular chondrocyte phenotype. We showed that in both mono- and co-cultures PRP significantly induced chondrogenic markers expression (Sox-9, Aggrecan, Versican, Col-II) in mRNA level. In protein level and ECM secretion, both PRP treatments and the co-ConvDiff appeared to deposit high amounts of GAG and Col-II. Although PRP had down-regulatory effects on hypertrophic markers, in mRNA level (Runx-2 and Col-X), when it came to protein, co-ConvDiff showed the lowest Col-X production. In the same way, the co-groups (co-ConvDiff and co-PRP) had lowered ALP activity compared to mono-groups, while PRP had increased calcium deposition compared to ConvDiff group. Regarding pathologic proinflammatory and pro-angiogenic markers, we observed that in comparison to conventional chondrogenic regimens, PRP is not advised since the co-ConvDiff group showed the lowest TNFα secretion with no stimulatory effects on VEGF production. Though the molecular aspects concerning PRP mechanisms of action and its crosstalk with MSC and AC cells were not addressed and are generally unanswered, our data showed that all the positive chondro-promotive effects of PRP and hypertrophy-suppressive effects of co-cultures do not necessarily act in synergy, and thus we cannot provide firm recommendations as to which regimen would work optimally for cartilage cell therapy. Nevertheless our data showed that PRP acted stronger in chondrogenesis while causing some calcium deposition and pro-angiogenic effects as well. While co-culture was more privileged for ALP and Col-X suppression and diminishing TNFα production. In spite of striking reduction in mRNA level of hypertrophic markers, the combinatory approach (co-PRP) could not fully attenuate hypertrophic maturation. Nevertheless, in a clinical point of view PRP might seem more appealing than conventional chondrogenic approach, since it costs less and can safely be provided autologously with significant chondro-promotive effects. Collectively, we observed synergistic effects for PRP and AC:MSC co-culture in enhancing chondrogenesis. Also, inclusion of AC could reduce some hypertrophic markers and angiogenic potential in PRP treated groups. We thus propose that the combination of PRP and co-culture would favor chondrogenesis while alleviate but not totally eradicate undesired hypertrophic and pathologic responses.

## Acknowledgements

This work has been partly funded by University of Tehran and partly by Iranian Council for Stem Cell Science and Technology.

## Conflict of interests

The authors declare that they have no conflict of interest.

## Figures and Tables

**Table 1:**
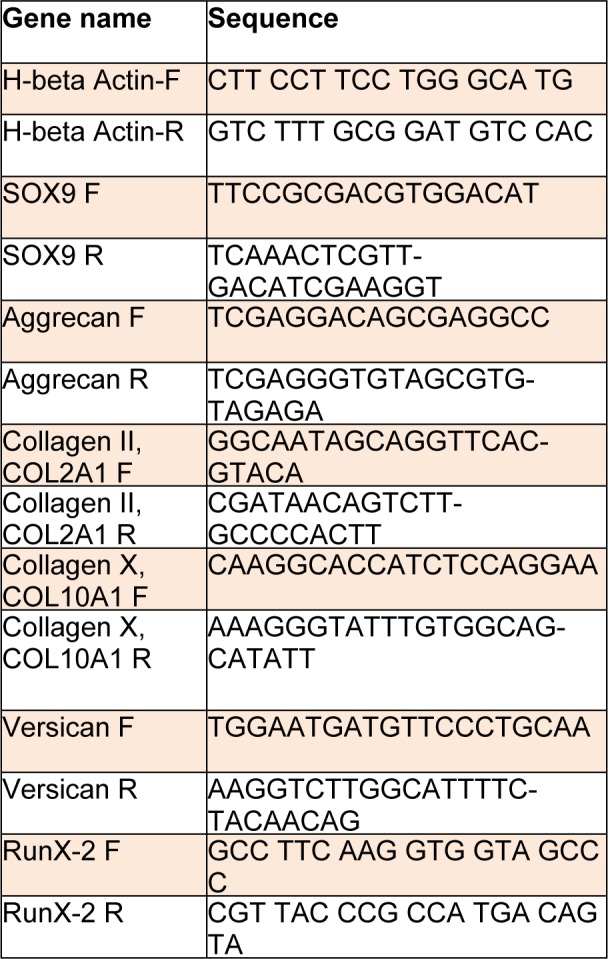
Primer sequences

## References

[1] Acharya C, Adesida A, Zajac P, Mumme M, Riesle J, Martin I (2012). Enhanced chondrocyte proliferation and mesenchymal stromal cells chondrogenesis in coculture pellets mediate improved cartilage formation. J Cell Physiol.

[2] Ahern BJ, Parvizi J, Boston R, Schaer TP (2009). Preclinical animal models in single site cartilage defect testing: a systematic review. Osteoarthr Cartilage.

[3] Ahmed N, Dreier R, Göpferich A, Grifka J, Grässel S (2007). Soluble signalling factors derived from differentiated cartilage tissue affect chondrogenic differentiation of rat adult marrow stromal cells. Cell Physiol Biochem.

[4] Akeda K, An HS, Okuma M, Attawia M, Miyamoto K, Thonar EJMA (2006). Platelet-rich plasma stimulates porcine articular chondrocyte proliferation and matrix biosynthesis. Osteoarthr Cartilage.

[5] Andia I, Maffulli N (2013). Platelet-rich plasma for managing pain and inflammation in osteoarthritis. Nat Rev Rheumatol.

[6] Anitua E, Zalduendo M, Prado R, Alkhraisat M, Orive G (2015). Morphogen and proinflammatory cytokine release kinetics from PRGF‐Endoret fibrin scaffolds: Evaluation of the effect of leukocyte inclusion. J Biomed Mater Res A.

[7] Babur BK, Kabiri M, Klein TJ, Lott WB, Doran MR (2015). The rapid manufacture of uniform composite multicellular-biomaterial micropellets, their assembly into macroscopic organized tissues, and potential applications in cartilage tissue engineering. PloS One.

[8] Becher C, Driessen A, Hess T, Longo UG, Maffulli N, Thermann H (2010). Microfracture for chondral defects of the talus: Maintenance of early results at midterm follow-up. Knee Surg Sports Traumatol Arthrosc.

[9] Bedi A, Feeley BT, Williams RJ (2010). Management of articular cartilage defects of the knee. J Bone Joint Surg A.

[10] Bian L, Zhai DY, Mauck RL, Burdick JA (2011). Coculture of human mesenchymal stem cells and articular chondrocytes reduces hypertrophy and enhances functional properties of engineered cartilage. Tissue Eng A.

[11] Böhme K, Winterhalter KH, Bruckner P (1995). Terminal differentiation of chondrocytes in culture is a spontaneous process and is arrested by transforming growth factor-β2 and basic fibroblast growth factor in synergy. Exp Cell Res.

[12] Boswell SG, Cole BJ, Sundman EA, Karas V, Fortier LA (2012). Platelet-rich plasma: A milieu of bioactive factors. Arthrosc - J Arthrosc Related Surg.

[13] Cooke M, Allon A, Cheng T, Kuo A, Kim H, Vail T (2011). Structured three-dimensional co-culture of mesenchymal stem cells with chondrocytes promotes chondrogenic differentiation without hypertrophy. Osteoarthr Cartilage.

[14] Dickhut A, Pelttari K, Janicki P, Wagner W, Eckstein V, Egermann M (2009). Calcification or dedifferentiation: requirement to lock mesenchymal stem cells in a desired differentiation stage. J Cell Physiol.

[15] Drengk A, Zapf A, Stürmer EK, Stürmer KM, Frosch K-H (2009). Influence of platelet-rich plasma on chondrogenic differentiation and proliferation of chondrocytes and mesenchymal stem cells. Cells Tissues Organs.

[16] Duffy GP, Ahsan T, O'Brien T, Barry F, Nerem RM (2009). Bone marrow–derived mesenchymal stem cells promote angiogenic processes in a time-and dose-dependent manner in vitro. Tissue Eng A.

[17] Farrell E, Both SK, Odörfer KI, Koevoet W, Kops N, O'Brien FJ (2011). In-vivo generation of bone via endochondral ossification by in-vitro chondrogenic priming of adult human and rat mesenchymal stem cells. BMC Musculoskel Dis.

[18] Filardo G, Kon E, Pereira Ruiz MT, Vaccaro F, Guitaldi R, Di Martino A (2012). Platelet-rich plasma intra- articular injections for cartilage degeneration and osteoarthritis: Single- versus double-spinning approach. Knee Surg Sports Traumatol Arthrosc.

[19] Fischer J, Dickhut A, Rickert M, Richter W (2010). Human articular chondrocytes secrete parathyroid hormone–related protein and inhibit hypertrophy of mesenchymal stem cells in coculture during chondrogenesis. Arthritis Rheum.

[20] Gerber H-P, Vu TH, Ryan AM, Kowalski J, Werb Z, Ferrara N (1999). VEGF couples hypertrophic cartilage remodeling, ossification and angiogenesis during endochondral bone formation. Nat Med.

[21] Hudgens JL, Sugg KB, Grekin JA, Gumucio JP, Bedi A, Mendias CL (2016). Platelet-rich plasma activates proinflammatory signaling pathways and induces oxidative stress in tendon fibroblasts. Am J Sports Med.

[22] Kabiri M, Lott WB, Kabiri E, Russell PJ, Doran MR (2013). In vitro assessment of migratory behavior of two cell populations in a simple multichannel microdevice. Processes.

[23] Kilroy GE, Foster SJ, Wu X, Ruiz J, Sherwood S, Heifetz A (2007). Cytokine profile of human adipose‐derived stem cells: Expression of angiogenic, hematopoietic, and pro‐inflammatory factors. J Cell Physiol.

[24] Kon E, Filardo G, Delcogliano M, Fini M, Salamanna F, Giavaresi G (2010). Platelet autologous growth factors decrease the osteochondral regeneration capability of a collagen-hydroxyapatite scaffold in a sheep model. BMC Musculoskelet Disord.

[25] Lee HR, Park KM, Joung YK, Park KD, Do SH (2012). Platelet-rich plasma loaded hydrogel scaffold enhances chondrogenic differentiation and maturation with up-regulation of CB1 and CB2. J Control Release.

[26] Malhotra A, Pelletier MH, Yu Y, Walsh WR (2013). Can platelet-rich plasma (PRP) improve bone healing? A comparison between the theory and experimental outcomes. Arch Orthop Traum Surg.

[27] Mamidi MK, Das AK, Zakaria Z, Bhonde R (2016). Mesenchymal stromal cells for cartilage repair in osteoarthritis. Osteoarthr Cartilage.

[28] Marquass B, Schulz R, Hepp P, Zscharnack M, Aigner T, Schmidt S (2011). Matrix-associated implantation of predifferentiated mesenchymal stem cells versus articular chondrocytes: In vivo results of cartilage repair after 1 year. Am J Sports Med.

[29] Maumus M, Manferdini C, Toupet K, Peyrafitte J-A, Ferreira R, Facchini A (2013). Adipose mesenchymal stem cells protect chondrocytes from degeneration associated with osteoarthritis. Stem Cell Res.

[30] Mifune Y, Matsumoto T, Takayama K, Ota S, Li H, Meszaros LB (2013). The effect of platelet-rich plasma on the regenerative therapy of muscle derived stem cells for articular cartilage repair. Osteoarthr Cartilage.

[31] Nagai T, Sato M, Kutsuna T, Kokubo M, Ebihara G, Ohta N (2010). Intravenous administration of anti- vascular endothelial growth factor humanized monoclonal antibody bevacizumab improves articular cartilage repair. Arthritis Res Ther.

[32] O'Sullivan J, D'Arcy S, Barry FP, Murphy JM, Coleman CM (2011). Mesenchymal chondroprogenitor cell origin and therapeutic potential. Stem Cell Res Ther.

[33] Pakfar A, Irani S, Hanaee-Ahvaz H (2017). Expressions of pathologic markers in PRP based chondrogenic differentiation of human adipose derived stem cells. Tissue Cell.

[34] Patel S, Dhillon MS, Aggarwal S, Marwaha N, Jain A (2013). Treatment with platelet-rich plasma is more effective than placebo for knee osteoarthritis a prospective, double-blind, randomized trial. Am J Sports Med.

[35] Pelttari K, Winter A, Steck E, Goetzke K, Hennig T, Ochs BG (2006). , Premature induction of hypertrophy during in vitro chondrogenesis of human mesenchymal stem cells correlates with calcification and vascular invasion after ectopic transplantation in SCID mice. Arthritis Rheum.

[36] Shafiee A, Kabiri M, Ahmadbeigi N, Yazdani SO, Mojtahed M, Amanpour S (2011). Nasal septum-derived multipotent progenitors: a potent source for stem cell-based regenerative medicine. Stem Cells Dev.

[37] Shafiee A, Kabiri M, Langroudi L, Soleimani M, Ai J (2016). Evaluation and comparison of the in vitro characteristics and chondrogenic capacity of four adult stem/progenitor cells for cartilage cell-based repair. J Biomed Mat Res A.

[38] Shen J, Li S, Chen D (2014). TGF-β signaling and the development of osteoarthritis. Bone research.

[39] Smyth NA, Fansa AM, Murawski CD, Kennedy JG (2012). Platelet-rich plasma as a biological adjunct to the surgical treatment of osteochondral lesions of the talus. Techniq Foot Ankle Surg.

[40] Smyth NA, Murawski CD, Fortier LA, Cole BJ, Kennedy JG (2013). Platelet-rich plasma in the pathologic processes of cartilage: Review of basic science evidence. Arthrosc - J Arthrosc Related Surg.

[41] Spaková T, Rosocha J, Lacko M, Harvanová D, Gharaibeh A (2012). Treatment of knee joint osteoarthritis with autologous platelet-rich plasma in comparison with hyaluronic acid. Am J Phys Med Rehabil.

[42] Spreafico A, Chellini F, Frediani B, Bernardini G, Niccolini S, Serchi T (2009). Biochemical investigation of the effects of human platelet releasates on human articular chondrocytes. J Cell Biochem.

[43] Wojdasiewicz P, Poniatowski ŁA, Szukiewicz D (2014). The role of inflammatory and anti-inflammatory cytokines in the pathogenesis of osteoarthritis. Mediat Inflamm.

[44] Wu L, Prins HJ, Helder MN, van Blitterswijk CA, Karperien M (2012). Trophic effects of mesenchymal stem cells in chondrocyte co-cultures are independent of culture conditions and cell sources. Tissue Eng Part A.

[45] Xie X, Zhang C, Tuan RS (2014). Biology of platelet-rich plasma and its clinical application in cartilage repair. Arthritis Res Ther.

[46] Yuan T, Guo S-C, Han P, Zhang C-Q, Zeng B-F (2012). Applications of leukocyte-and platelet-rich plasma (L- PRP) in trauma surgery. Curr Pharm Biotechnol.

[47] Zhu Y, Yuan M, Meng H, Wang A, Guo Q, Wang Y (2013). Basic science and clinical application of platelet- rich plasma for cartilage defects and osteoarthritis: a review. Osteoart Cartilage.

